# EFHD1 Activates SIK3 to Limit Colorectal Cancer Initiation and Progression via the Hippo Pathway

**DOI:** 10.7150/jca.103229

**Published:** 2025-01-20

**Authors:** Qionghui Huang, Xiaoyan Tang, Caiyan Gan, Qiaoting Deng, Shaobin Zhi, Qingyan Huang, Xiaoqi Zheng, Xueqiong Li, Zengfeng Pan, Mingfeng Huang

**Affiliations:** 1 Institute of Cardiovascular Disease, Meizhou People's Hospital, Meizhou Academy of Medical Sciences, Meizhou, China.; 2 GuangDong Engineering Technological Research Center of Molecular Diagnosis in Cardiovascular Diseases, Meizhou, China.; 3 Institute of basic medical sciences, Meizhou People's Hospital, Meizhou Academy of Medical Sciences, Meizhou, China.; 4 Medical College of Jiaying University, Meizhou, China.

**Keywords:** Colorectal cancer, EFHD1, SIK3, EMT, Hippo pathway

## Abstract

Colorectal cancer (CRC) is one of the most commonly diagnosed cancers, with high rates of metastasis and lethality. EF-hand domain-containing protein D1 (EFHD1) and salt-inducible kinase 3 (SIK3) have been studied in several cancer types. Aberrant expression of EFHD1 and SIK3 has been observed in CRC, but little research has addressed their regulatory abilities and signaling pathways. In this study, we aimed to explore the efficacy of EFHD1 in inhibiting CRC proliferation and metastasis and to elucidate the underlying mechanisms involved in the upregulation of SIK3 expression. Cell viability, colony formation, wound healing, Transwell assay, orthotopic xenograft, and pulmonary metastasis mouse models were used to detect the antiproliferative and anti-metastatic effects of EFHD1 against CRC *in vitro* and *in vivo*. The Gene Expression Profiling Interactive Analysis (GEPIA) database was used to determine EFHD1 and SIK3 expression in CRC. The regulatory roles of EFHD1 and SIK3 in mediating anti-metastatic effects in CRC were measured using western blotting, immunohistochemical, and immunofluorescence analyses. The results showed that EFHD1 expression was significantly repressed in the clinical CRC samples. EFHD1 markedly suppressed cell proliferation, migration, and invasion *in vitro* and inhibited tumor growth and metastasis *in vivo*. Analysis of the GEPIA database revealed that EFHD1 expression positively correlated with SIK3 expression. SIK3 overexpression inhibited the migration of CRC cells, and SIK3 knockdown partially eliminated the inhibitory effects of EFHD1 on CRC metastasis. EFHD1 exerted anti-metastatic effects against CRC via upregulating SIK3 and inhibiting epithelial-mesenchymal transition (EMT) processing through modulating the Hippo signaling pathway. Collectively, these findings identify EFHD1 as a potent SIK3 agonist and highlight the EFHD1-SIK3 axis as a key modulator of the Hippo signaling pathway in CRC. EFHD1 serves as a novel regulator and is worthy of further development as a novel therapeutic target in CRC.

## Introduction

Colorectal cancer (CRC) is a common type of cancer that starts in the mucosal epithelium of the colon and rectum, encompassing malignant tumors in the ascending colon, transverse colon, descending colon, sigmoid colon, and rectum. While the exact causes of CRC are not yet fully understood, potential links include genetic, dietary, environmental, and lifestyle factors 1. Although there have been significant advances in early detection, mostly through proctoscopy, colonoscopy, and imaging tests, CRC remains the second deadliest malignancy globally, with patients facing high rates of recurrence and metastasis 2. The metastasis of CRC cells is a rapid process that mostly involves lymph node metastasis, blood metastasis, direct infiltration, and implantation. Fifty percent of patients are diagnosed with metastatic CRC, and the 5-year overall survival rate is only 14% 3. Therefore, there is a critical need for innovative therapies to slow the progression of metastatic CRC.

In recent decades, targeting certain biomarkers has proven to be an effective way of providing precision medicine to patients. The mutant biomarkers identified in CRC include *KRAS, NRAS, BRAF,* and* PIK3CA* 4. Abnormal *KRAS, NRAS, BRAF,* or* PIK3CA* expression disrupts regular cellular activities associated with tumor formation, proliferation, migration, spread, and angiogenesis. Although medications that target these genes significantly improve the progression-free survival rate of patients with CRC, the overall prognosis remains dismal, and there is no particular treatment for patients with wild-type CRC 5. Hence, an increasing number of researchers are dedicated to identifying more viable biomarkers and investigating potential molecular pathways for metastatic CRC to develop new strategies for its treatment.

EF-hand domain-containing protein D1 (EFHD1), also known as swiprosin-2, is an important member of the EF-hand superfamily that regulates calcium-binding and transport. EFHD1 consists of a disordered N-terminal region, proline-rich elements, two EF-hands, and a C-terminal coiled-coil domain, and is engaged in a variety of cellular processes, such as mitosis, axonal morphogenesis, and cytoskeletal rearrangement 6. Recent studies have indicated that EFHD1 is associated with tumorigenesis. For example, previous genomic studies have validated that EFHD1 is highly expressed in breast cancer 7, making it a promising target for tumor prediction. Compared to non-cancerous tissue samples, EFHD1 expression increases dramatically in stomach cancer tissue samples, leading to unfavorable clinical outcomes 8. Additionally, the aberrant expression of EFHD1 has been observed in clear-cell renal cell carcinoma. EFHD1 inhibits cell migration and invasion *in vitro* and tumor metastasis *in vivo* by modulating the Hippo signaling pathway 9. However, few studies have investigated the anti-cancer effects of EFHD1 in CRC. Salt-inducible kinase 3 (SIK3), an important SIK subtype, belongs to the AMP-activated protein kinase family and exhibits serine/threonine kinase activity 10. SIK3 is constitutively expressed in tissues and is ubiquitous in humans 11. As a cell cycle regulator, SIK3 controls cell growth and development by stimulating the Hippo signaling pathway 12. However, the dysfunction of SIK3 has been identified in various types of cancers. High expression of SIK3 promotes cell proliferation by regulating mTOR and cell migration via the activation of the epithelial-mesenchymal transition (EMT) process in breast cancer 13, 14. Moreover, SIK3 ​can protect pancreatic cancer cells from cytotoxic T-cell attack by triggering TNF-induced NF-κB translocation 15. Different from breast and pancreatic cancers, SIK3 serves as a tumor suppressor in ovarian and lung cancers. SIK3 can increase the chemotherapeutic efficacy of Taxol plus cisplatin treatment and the overall survival rate of patients with ovarian cancer 16. While the loss of SIK3 facilitates tumor progression in non-small cell lung cancer 17, few studies have addressed the regulatory role of SIK3 in CRC.

According to various Gene Expression Profiling Interactive Analysis (GEPIA) datasets, the expression of EFHD1 and SIK3 are significantly higher in normal tissues than in tumor tissues of patients with CRC, and there is a strong positive correlation between EFHD1 and SIK3 in both normal colon tissues and CRC tumor tissues. Based on these findings, we speculated that EFHD1 may exert anti-cancer effects in CRC by regulating SIK3 expression. In the present study, we explored the anti-proliferative and anti-metastatic effects of EFHD1, along with the role of SIK3 in mediating EFHD1's anti-neoplastic effects through the modulation of the Hippo signaling pathway.

## Materials and methods

### Human CRC tissue microarray (TMA) slide

Human CRC TMA slides (CRC-2111, *n* = 20) were purchased from Service Bio (Wuhan, China). EFHD1 expression on these slides was detected using immunohistochemical (IHC) analysis with an EFHD1 polyclonal antibody (1:50, Cat # PA5-61934; Invitrogen).

### Clinical samples and cell lines

Ten patients diagnosed with CRC at the Meizhou People's Hospital in 2024 were randomly chosen for this study. Human CRC and adjacent normal tissue samples were surgically collected from these patients. The selection criteria were as follows: (1) pathological diagnosis of CRC, (2) age 18-85 years, and (3) complete surgical resection. This study was approved by the Institutional Research Ethics Committee of Meizhou People's Hospital.

CRC cell lines HCT116, SW480, HT29, and Caco2 were purchased from ATCC (Manassas, VA, USA). The normal human colonic epithelial cell line NCM460 was obtained from Otwo Biotech (Shenzhen, China). Cells were maintained in FBS-containing Dulbecco's Modified Eagle's Medium (DMEM; Gibco, USA) under humidified conditions (37 °C, 5% CO_2_).

### Establishment of transfected cell lines using lentivirus or plasmid

To obtain cell lines with stable overexpression or silencing of EFHD1 or SIK3, HCT116 and SW480 cells were infected with the following viral constructs: OE-EFHD1 (sc-413376-LAC; Santa Cruz, USA), OE-SIK3 (sc-406729-LAC; Santa Cruz, USA), OE-Control (sc-108084; Santa Cruz, USA) for overexpression, as well as sh-EFHD1 (sc-94294-V; Santa Cruz, USA) and sh-Control (sc-108080; Santa Cruz, USA) for gene silencing. Additionally, siRNA plasmids, including si-SIK3 (sc-97056; Santa Cruz, USA) and si-Control (sc-37007, Santa Cruz, USA), were transfected to achieve transient silencing of SIK3. The efficiency of EFHD1 and SIK3 overexpression or knockdown was confirmed through PCR and western blot analyses.

### Cell viability assay

Cell viability was measured using the MTT assay. Transfected HCT116 and SW480 cells were seeded for 24 h or 48 h and then treated with MTT and DMSO. The absorbance was measured at 490 nm using a microplate reader (Thermo Fisher Scientific), and the 50% inhibitory concentration (IC_50_) values were calculated.

### Colony formation assay

For the colony formation assay, 500 viable HCT116 or SW480 cells were cultured in six-well plates with the medium refreshed every 3 d for cell colonization. After 2 weeks, the cells were fixed with 95% ethanol and stained with 0.1% crystal violet staining solution. Images of cell colonies were acquired using a camera (Canon, Japan).

### Wound healing assay

To assess cell motility, HCT116 and SW480 cells (5 × 10^5^ cells) were seeded in 24-well plates and cultured until achieving 95% confluence. A straight scratch was gently made to inflict a wound, and images were taken at 0 and 24 h under an optical microscope (Leica, Germany).

### Cell migration and invasion assay

The cell migration and invasion assays were both conducted using 24-well Transwell insert chambers (8 μm, Millicell, Germany). HCT116 and SW480 cells (5 × 10^4^) were plated in the upper chamber coated with or without Matrigel™ (BD Biosciences, Sparks, MD, USA), and DMEM containing 20% FBS was added to the lower compartment. After 24 h, cells on the lower surface were fixed, stained, and scored under a light microscope (Leica, Germany).

### RNA extraction and quantitative real-time PCR

Total RNA from cells was extracted before quantification by measuring the ratio of absorbance between 260-280 nm (purity 1.9-2.1). EFHD1 RNA levels were quantified using specific primers (F: 5'-GGTGTCAAAGGTGCCAAGAACTTC-3', R: 5'-CTCCTCCTCCTCCCGCTTCC-3') and normalized to GAPDH expression (F: 5'-CACCCACTCCTCCACCTTTGAC-3', R: 5'-GTCCACCACCCTGTTGCTGTAG-3'). RNA quantification followed the standard protocol for the PrimeScript™ RT reagent Kit and SYBR premix EX Taq™ Kit (TakaRa Biotechnology, Japan).

### Animal experiment studies

Male BALB/c nude mice (6-weeks-old) were obtained from Guangdong Zhi Yuan Laboratory Animal Technology Co., Ltd., where they were maintained under specific-pathogen-free conditions. All animal experiments were conducted in accordance with the International Ethics Guidelines and the National Institutes of Health Guidelines for the Care and Use of Laboratory Animals. Mice were maintained under a 12-hour light/dark cycle with sterile food and water. The care and use of the animals complied with the institutional guidelines.

To construct the CRC orthotopic xenograft mouse model, HCT116-OE-EFHD1-luc, HCT116-OE-Control-luc, HCT116-sh-EFHD1-luc, or HCT116-sh-Control-luc cells (5×10^6^ cells/mL) were suspended in cold phosphate buffered saline and injected subcutaneously into the right dorsal flank of the mice. When the CRC tumor in the subcutaneous tissue was macroscopically visible (volume < 1.5 cm^3^), the tumors were isolated, cut into 1 mm^3^ sections, and embedded into the mesentery of the mice. Fluorescence radiance was detected and recorded weekly using a Carestream *In Vivo* Imaging System (Perkin Elmer). After 4 weeks, the mice were sacrificed under anesthesia, and the primary tumors in the colon were excised.

To establish tail vein lung metastasis mouse models, a suspension of HCT116-OE-EFHD1-luc, HCT116-OE-Control-luc, HCT116-sh-EFHD1-luc, or HCT116-sh-Control-luc cells (1 × 10^7^ cells in 200 μL) were intravenously injected into each nude mouse (*n* = 8 in each group). Mice were imaged once a week, and lung tissues were collected after 4 weeks.

### Western blotting analysis

Cellular proteins and CRC human tissues were collected, and their concentrations were tested. A certain amount of protein (30 μg) from each sample was separated and transferred to PVDF membranes. The membranes were then blocked and incubated with 1:500 of EFHD1 (H00080303; Abnova), p-LATS1 (8654; CST), p-YAP1 (13008; CST), p-TAZ (59971; CST), TEAD1 (ab133533; Abcam), 1:1,000 of E-cadherin (A3044; ABclonal), N-cadherin (A19083; ABclonal), Vimentin (A19607; ABclonal), MMP2 (A6247; ABclonal), MMP9 (A0289; ABclonal), MCM2 (ab108935; Abcam), PCNA (ab92552; Abcam), LATS1 (3477; CST), YAP1 (ab205270; Abcam), TAZ (83669; CST), SIK3 (ab227044; Abcam), GAPDH (ab9485; Abcam), and H3 (ab1791; Abcam) antibodies overnight. Subsequently, membranes were incubated with 1:3,000 secondary antibodies, and proteins were detected with ECL reagent using a western blot detection system (Bio-Rad, Singapore).

### Histopathological and IHC analysis

For histopathological analysis, the tissues were fixed, dehydrated, and embedded in paraffin wax, then cut into 5-μm sections and stained with hematoxylin and eosin (H&E). Histological characterization was performed using an inverted microscope (Nikon, Tokyo, Japan).

For immunohistochemistry (IHC) analysis, after undergoing a routine antigen retrieval process using sodium citrate buffer (pH 6), the paraffin slides were incubated with 1:50 EFHD1 overnight. The slides were then incubated with 1:200 secondary antibodies and stained using a DAB Advanced Chromogenic Kit (Invitrogen). Finally, images were captured using an inverted microscope (Nikon, Tokyo, Japan).

### Immunofluorescent (IF) staining

For immunofluorescence (IF) analysis, transfected cells were seeded and cultured with si-SIK3 or si-Control for 24 h. Next, the cells were fixed, permeabilized, and incubated with YAP1 (1:500) overnight. Slides were stained with Alexa Fluor594-conjugated secondary antibodies and DAPI. Images were captured using a fluorescence microscope (Zeiss).

### Statistical analysis

The difference between two groups was determined using the Student's t-test. Multiple comparisons were performed using a one-way analysis of variance. A *p*-value of <0.05 was considered statistically significant. Statistical analyses were performed using GraphPad Prism version 9.0.

## Results

### EFHD1 expression is decreased in CRC

**Figure 1:**
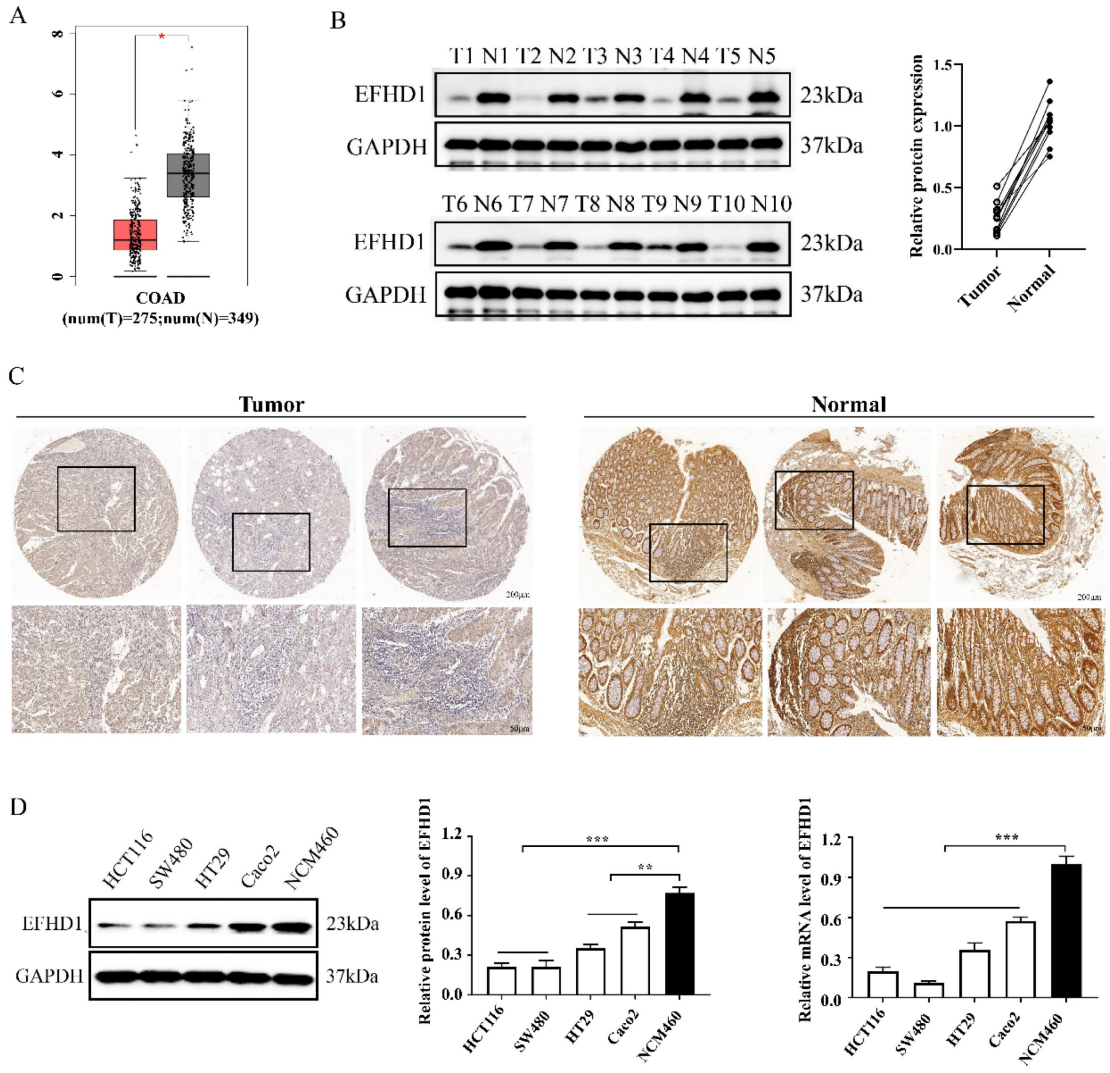
** (A)** Scatter diagram derived from gene expression data in GEPIA comparing the expression of EFHD1 in the tumor tissue and the normal tissue in CRC. **(B)** EFHD1 protein expression in the indicated cancer tissues (T) and their corresponding adjacent nontumoral tissues (N) using western blot assay. **(C)** Representative IHC images of EFHD1 expression in CRC tissues and their corresponding normal tissues. **(D)** EFHD1 expression in HCT116, SW480, HT29, Caco2 and NCM460 cells were detected by western blot and q-PCR.

To explore the clinical significance of EFHD1, GEPIA datasets and clinical CRC tissue samples were analyzed. Analysis of the GEPIA datasets showed that EFHD1 was less expressed in CRC tumor tissues than in normal tissues (**Figure 1A**). Western blotting and immunohistochemistry were performed to measure EFHD1 expression in paired fresh paracancerous and CRC tissue samples. The results showed that EFHD1 expression in paracancerous tissue samples was noticeably higher than that in tumor tissue samples (**Figure 1B, C**).

In addition, we compared EFHD1 expression between normal and CRC cells. The results indicated that the protein and mRNA levels of EFHD1 were significantly higher in the normal colonic epithelial cell line NCM460 than in the CRC (HCT116, SW480, HT29, and Caco2) cells (**Figure 1D**).

### EFHD1 inhibits CRC cell proliferation

**Figure 2:**
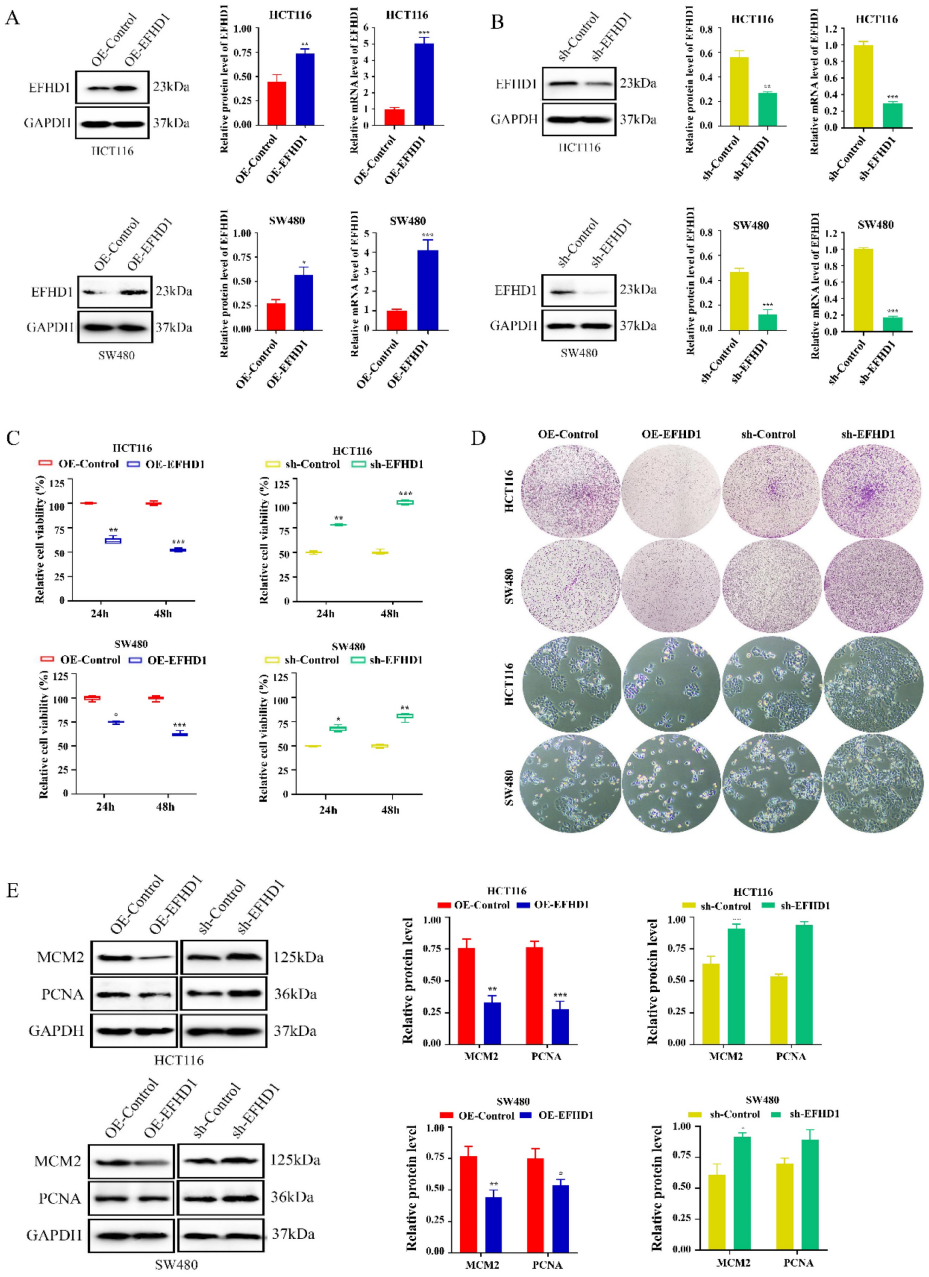
** EFHD1 suppresses the proliferation of CRC cells *in vitro*. (A-B)** The protein and mRNA levels of EFHD1 in HCT116 and SW480 cells stably overexpressing EFHD1 or silcencing EFHD1. **(C)** IC_50_ of HCT116 and SW480 cells stably overexpressing EFHD1 or silcencing EFHD1 for 48h. **(D)** The colony-formation and morphological changes of HCT116 and SW480 were monitored in HCT116 and SW480 cells stably overexpressing EFHD1 or silcencing EFHD1. **(E)** The protein levels of MCM2 and PCNA in HCT116 and SW480 cells stably overexpressing EFHD1 or silcencing EFHD1. Data were represented as mean ± SD. *p < 0.05, **p < 0.01 and ***p < 0.001 as compared to the control group.

To confirm the effects of EFHD1 on CRC growth, we established EFHD1 overexpression and knockdown CRC cell lines using EFHD1-lentivirus constructs (**Figure 2A, B**). The cytotoxic effects of EFHD1 on the HCT116 and SW480 CRC cell lines are shown in **Figure 2C**. The results indicated that EFHD1 overexpression markedly suppressed the proliferation of HCT116 and SW480 cells after 24 and 48 h. The results of the morphology and colony formation experiments also supported that EFHD1 overexpression had an inhibitory effect on cell survival (**Figure 2D**). However, EFHD1 knockdown significantly promoted the growth of HCT116 and SW480 cells (**Figure 2C, D**). Moreover, EFHD1 overexpression inhibited the cell proliferation index (MCM2 and PCNA) in HCT116 and SW480 cells, whereas EFHD1 knockdown had the opposite effect (**Figure 2E**), suggesting that EFHD1 has potent antiproliferative effects on CRC cells.

### EFHD1 suppresses CRC cell metastasis

**Figure 3:**
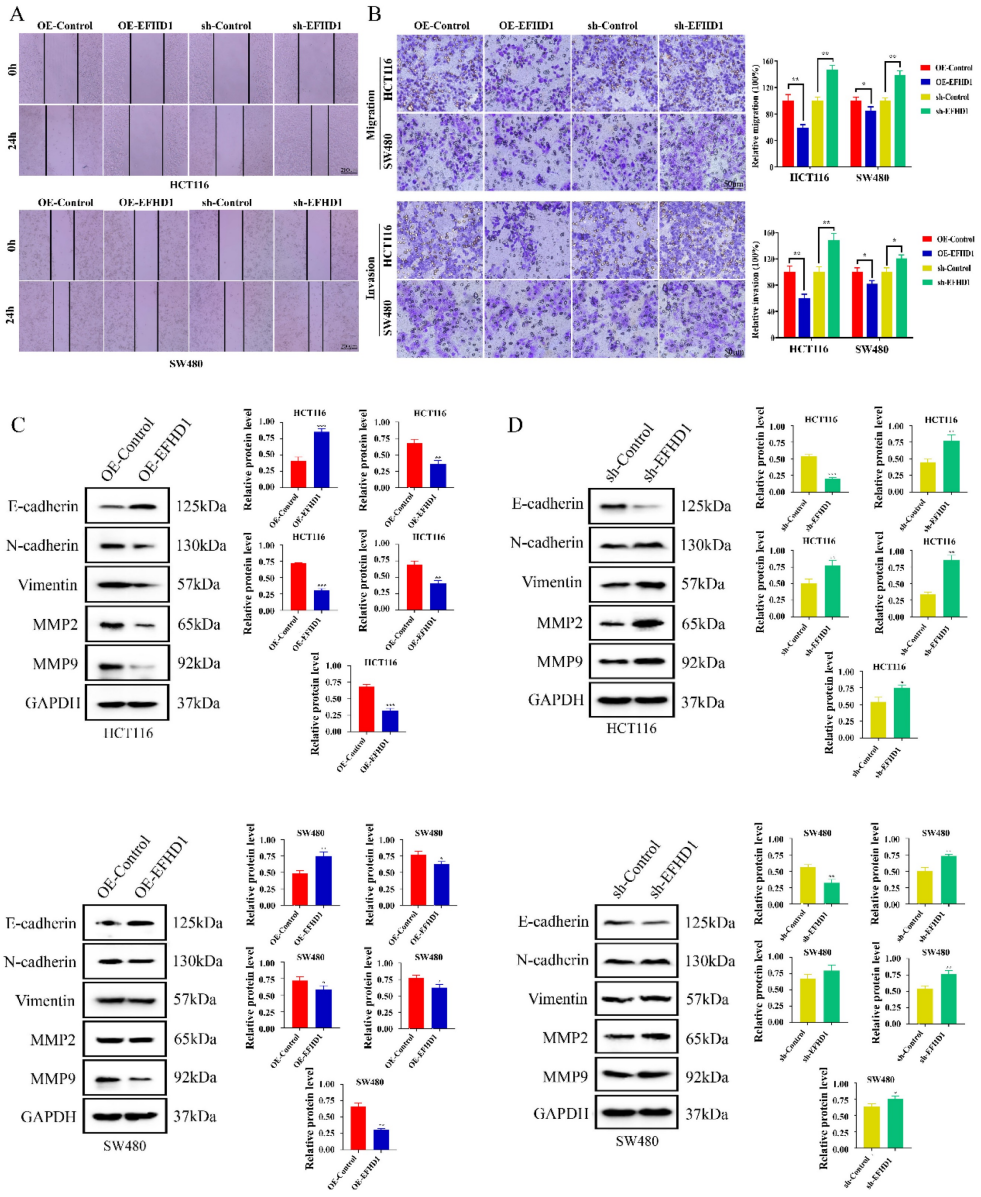
** EFHD1 inhibits the metastasis of CRC cells *in vitro*. (A)** Wound healing assay. A scratch was made in monolayers of HCT116 and SW480 cells transduced with overexpressing EFHD1 or silcencing EFHD1 for 24 h and monitored with an inverted microscope. **(B)** Cell migration and invasion transwell assays. HCT116 and SW480 cell suspension was plated in the upper chamber of transwell insert, and migrated or invaded cells were fixed and stained with 0.1% crystal violet of cells. Representative photographs are presented (left) and the relative number of migratory and invasive cells (right) was counted. **(C-D)** Western blot analysis. Protein levels of EMT-related proteins in HCT116 and SW480 cells transduced with overexpressing EFHD1 or silcencing EFHD1. GAPDH was used as a loading control. Data were represented as mean ± SD. *p < 0.05, **p < 0.01 and ***p < 0.001 as compared to the control group.

To investigate the effects of EFHD1 on CRC cell migration and invasion, wound healing and Transwell assays were performed. As shown in **Figure 3A**, the same “wounded” area was scratched and photographed at 0 h. After 24 h, EFHD1-silencing cells moved rapidly toward the middle blank space. However, EFHD1 overexpression significantly inhibited gap closure, suggesting that EFHD1 exerted substantial inhibitory effects on cell motility. In the Transwell assay, a large number of migrated and invaded cells were found in the EFHD1-silencing group, as compared to the control group. However, EFHD1 overexpression led to a significant reduction in the migratory and invasive capacities of the cells (**Figure 3B**). These results indicated that EFHD1 markedly inhibited the metastasis of CRC cells* in vitro*.

Metastasis-related proteins were examined to explore the mechanisms underlying the inhibitory effects of EFHD1 on metastasis. As shown in **Figure 3C** and **D**, low expression of EMT-processing markers, including N-cadherin, vimentin, MMP2, and MMP9, as well as high expression of E-cadherin, were found in the EFHD1-overexpressing group. Nevertheless, EFHD1 knockdown increased the levels of N-cadherin, vimentin, MMP2, and MMP9 while attenuating the level of E-cadherin in HCT116 and SW480 CRC cells. Taken together, these results indicate that EFHD1 suppresses CRC metastasis *in vitro* by regulating EMT.

### EFHD1 overexpression attenuated tumorigenesis in orthotopic CRC mouse models

**Figure 4:**
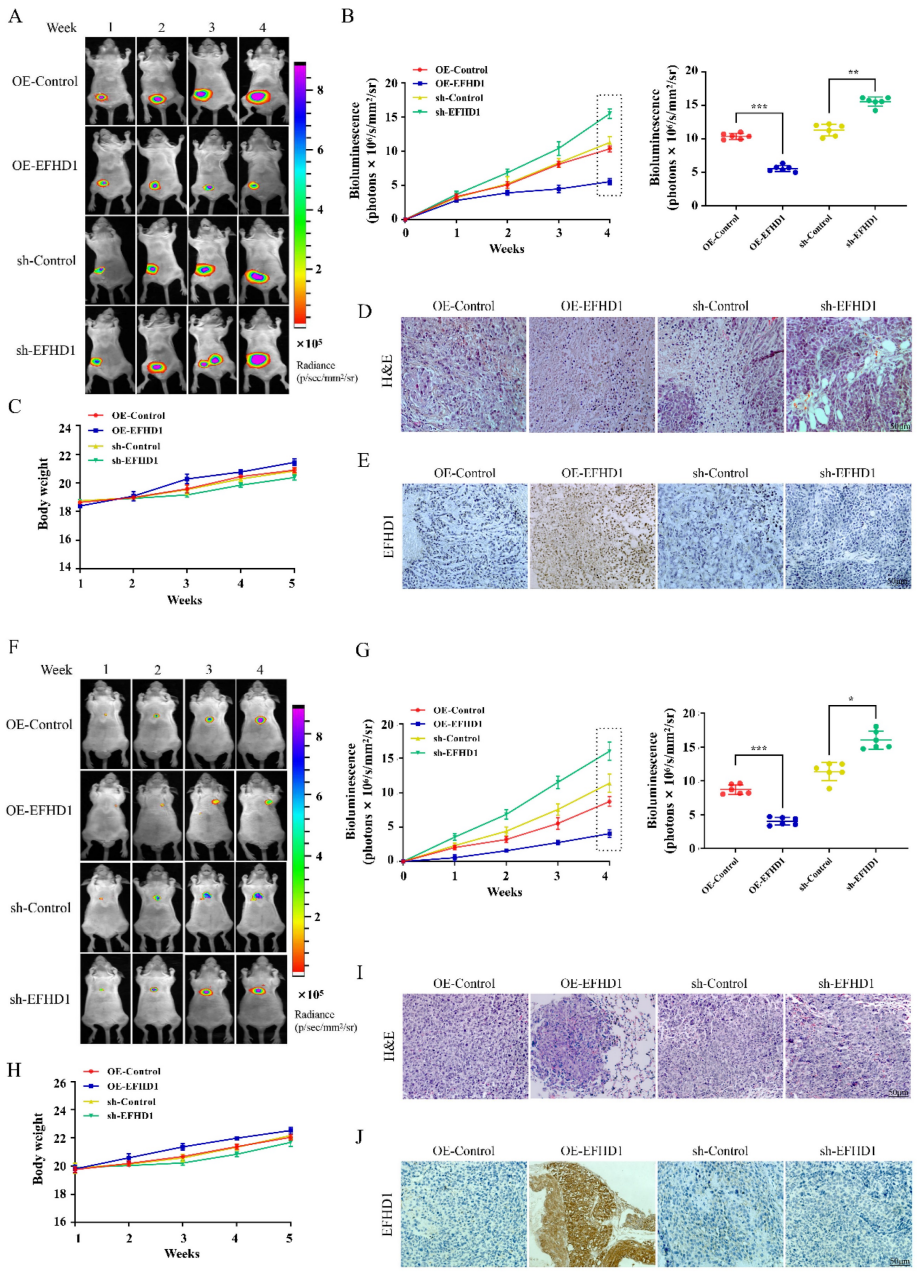
** EFHD1 limits the tumor growth and tumor metastasis in CRC nude mouse models. (A)** Bioluminescent imaging for HCT116-luc orthotopic xenograft colon tumors at different time points. **(B)** Bioluminescence signals recorded at the indicated time points were represented as total photon flux (left); Bioluminescence signals were analyzed on the 4^th^ week (right) in HCT116-luc orthotopic xenograft model. **(C)** The body weight was recorded every day of experimental animals throughout the study duration in HCT116-luc orthotopic xenograft model. **(D)** Analysis of tumor H & E staining in HCT116-luc orthotopic xenograft model. **(E)** Analysis of EFHD1 staining by IHC in HCT116-luc orthotopic xenograft model. **(F)** Bioluminescence imaging for HCT116-luc lung metastasis at different time points. **(G)** Bioluminescence signals recorded at the indicated time points were represented as total photon flux (left); Bioluminescence signals were analyzed on the 4^th^ week (right) in HCT116-luc lung metastasis model. **(H)** The body weight was recorded every day of experimental animals throughout the study duration in HCT116-luc lung metastasis model. **(I)** Analysis of lung H & E staining in HCT116-luc lung metastasis model. **(J)** Analysis of EFHD1 staining by IHC in HCT116-luc lung metastasis model. Data were represented as mean ± SD. *p < 0.05, **p < 0.01 and ***p < 0.001 as compared to the control group.

To study the anti-tumor potential of EFHD1, orthotopic CRC models were established by implanting luciferase-transfected HCT116 cells into the mesentery of mice. As shown in **Figure 4A**, fluorescence intensity was comparable across all groups during the first week following the transplantation of EFHD1-overexpressing or EFHD1-silencing HCT116 cells into nude mice. However, fluorescence intensity gradually increased in the EFHD1-silencing group, whereas it decreased in the EFHD1-overexpressing group relative to their respective controls. On the 5th week, there was a significant difference between the control group and EFHD1 treatment groups (**Figure 4B**). There were no significant differences in body weights between the control group and EFHD1 treatment group (**Figure 4C**). H&E staining of tumor sections revealed intact cellular structures and nuclei in both the control and EFHD1-silencing groups, while the EFHD1-overexpressing group exhibited pronounced tumor necrosis (**Figure 4D**). IHC analysis confirmed high EFHD1 expression in the EFHD1-overexpressing group and low expression in the EFHD1-silencing group (**Figure 4E**). These results indicate that EFHD1 markedly inhibits orthotopic CRC tumor growth.

### EFHD1 attenuates tumor metastasis* in vivo*

To study the anti-metastatic potential of EFHD1, pulmonary metastasis CRC animal models were established by tail vein injection of luciferase-transfected HCT116 cells overexpressing or silencing EFHD1. In the pulmonary metastasis mouse model, the fluorescence intensity gradually increased over time across all groups (**Figure 4F**). EFHD1 overexpression significantly slowed the progression of lung metastasis, whereas EFHD1 knockdown accelerated tumor metastasis. On the 4th week, there was a significant difference between the control group and EFHD1 treatment group (**Figure 4G**). However, there was no significant difference in body weight between the control group and EFHD1 treatment group (**Figure 4H**). H&E staining of lung tissues showed that the number of lung tumor nodules in the EFHD1-overexpressing group was markedly lower than that in the control group, whereas there were more tumor nodules in the EFHD1-silencing group (**Figure 4I**). IHC staining corroborated these findings, showing high EFHD1 expression in the EFHD1-overexpressing group and low expression in the EFHD1-silencing group (**Figure 4J**). Taken together, these results indicate that EFHD1 exhibits prominent anti-metastatic activity *in vivo*.

### SIK3 knockdown weakens the metastasis inhibitory effects of EFHD1

**Figure 5:**
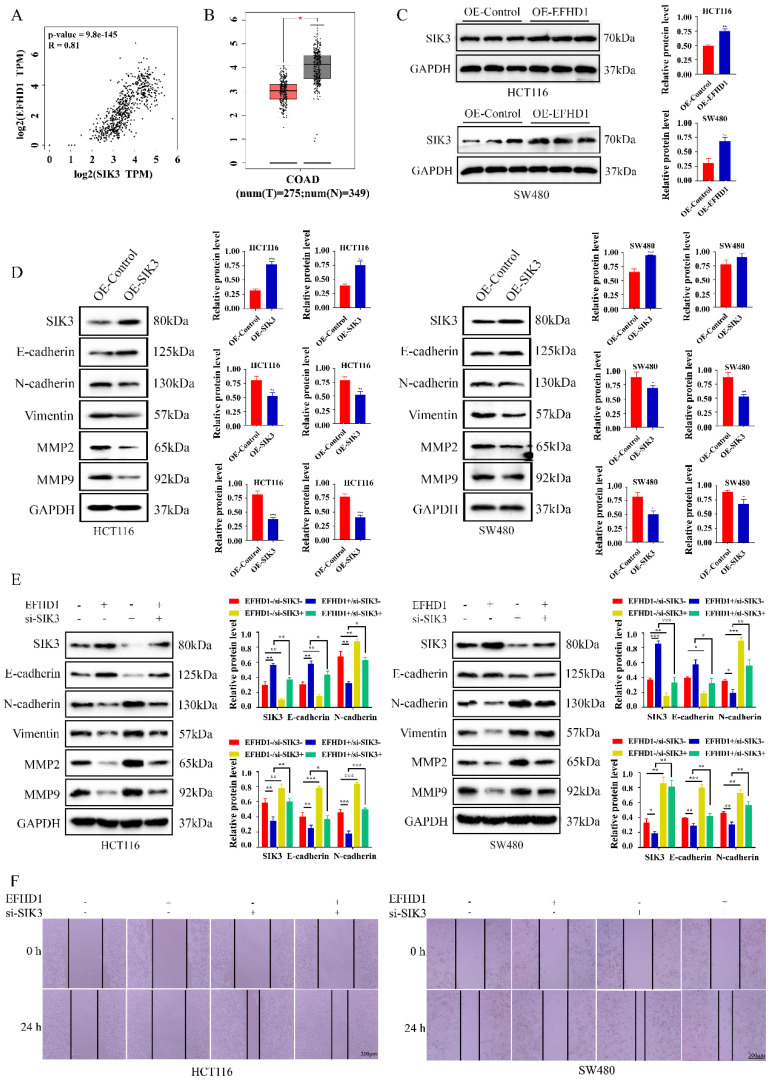
** Knockdown of SIK3 impairs the cell metastasis inhibitory effects of EFHD1 on CRC. (A)** Spearman correlation analysis between EFHD1 and SIK3 of normal colon and CRC tissues in GAPIA. **(B)** Scatter diagram derived from gene expression data in GEPIA comparing the expression of SIK3 in the tumor tissue and the normal tissue in CRC. **(C)** Protein expression and statistical analysis of SIK3 in HCT116 and SW480 cells transduced with overexpressing EFHD1.** (D)** Protein expression and statistical analysis of EMT-related proteins in HCT116 and SW480 cells transduced with overexpressing SIK3. **(E)** Western blot analysis of the related markers of EMT processing in EFHD1-overexpressed HCT116 and SW480 cells treated with or without SIK3-knockdown plasmid for 24 h. GAPDH was used as a loading control. **(F)** Wound healing assay. A scratch was made in monolayers of HCT116 and SW480 cells transduced with overexpressing EFHD1 and treated with or without SIK3-knockdown plasmid (magnification, 50x) and monitored with an inverted microscope. Data were represented as mean ± SD. *p < 0.05, **p < 0.01 and ***p < 0.001 as compared to the control group.

To examine the potential targets of EFHD1, gene expression correlations were analyzed using GEPIA. The results showed a strong positive correlation between EFHD1 and SIK3 in CRC tumors and normal tissues (**Figure 5A**). In addition, SIK3 expression was lower in CRC tumor tissues than in normal tissues (**Figure 5B**), and EFHD1 overexpression significantly increased the protein level of SIK3 (**Figure 5C**). Based on these findings, we hypothesized that SIK3 might have anti-CRC effects similar to those of EFHD1 and that it is regulated by EFHD1. As shown in **Figure 5D**, SIK3 overexpression upregulated E-cadherin expression and downregulated N-cadherin, vimentin, MMP2, and MMP9 expression in HCT116 and SW480 CRC cells. To determine whether EFHD1 exerted its tumor suppressor activity by regulating the expression of SIK3, a confirmatory experiment was conducted by overexpressing EFHD1 in cells with or without SIK3 knockdown. The results indicated that SIK3 knockdown increased the levels of N-cadherin, vimentin, MMP2, and MMP9 while decreasing the level of E-cadherin (**Figure 5E**). SIK3 knockdown partially abolished the inhibitory effects of EFHD1 on CRC metastasis in HCT116 and SW480 cells, as confirmed by a wound healing assay (**Figure 5F**). These results suggest that SIK3 knockdown promotes metastasis, whereas EFHD1 suppresses it by regulating SIK3 expression in CRC.

### EFHD1 regulated Hippo signaling pathway targeting SIK3

**Figure 6:**
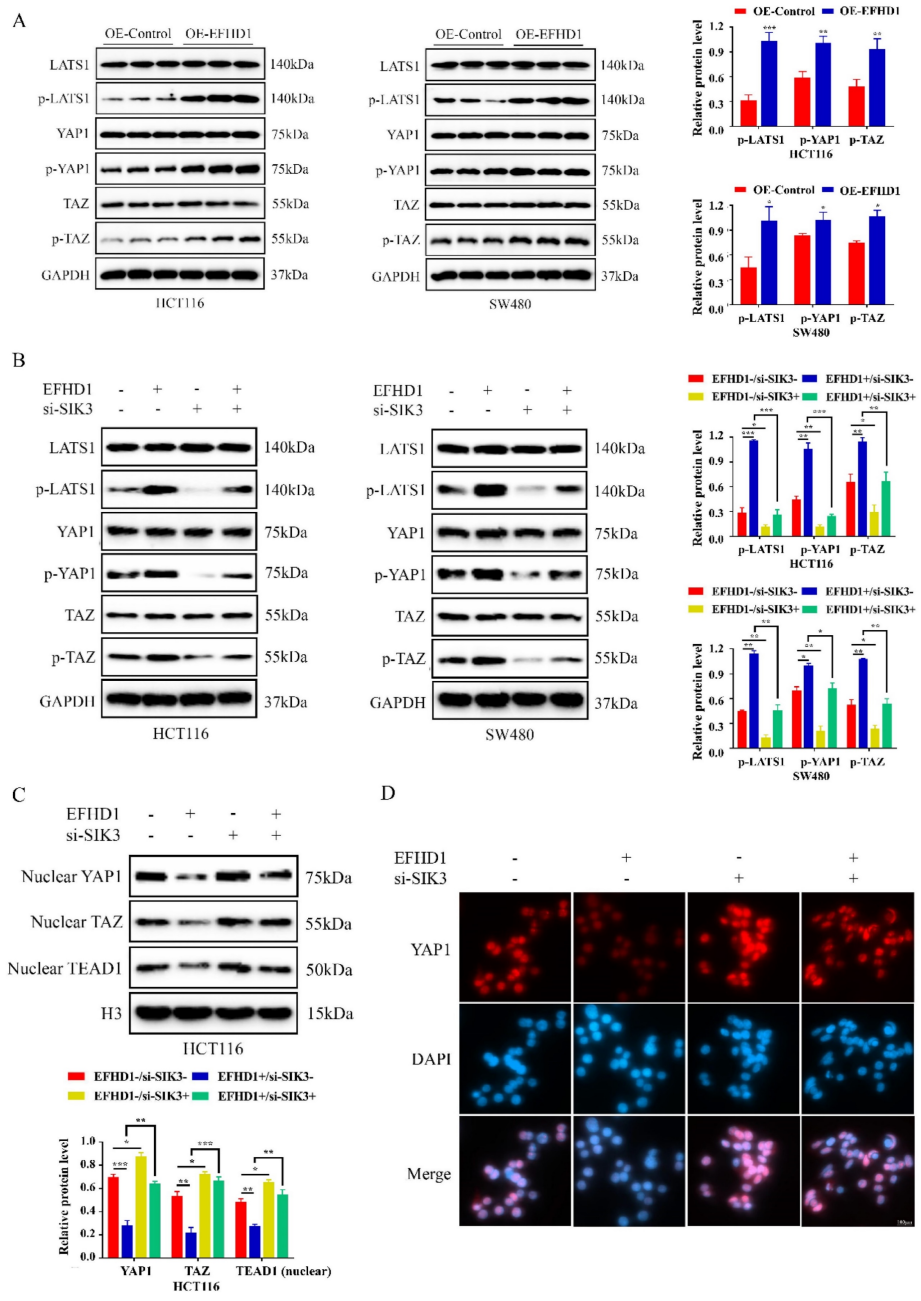
** EFHD1 modulates Hippo pathway to upregulate SIK3 expression. (A)** Western blot and statistical analysis of the specific protein expressions in EFHD1-overexpressed-transfected HCT116 and SW480 cells. GAPDH was used as a loading control. **(B)** Western blot and statistical analysis of the specific protein expressions in EFHD1-overexpressed-transfected HCT116 and SW480 cells in the presence or absence of SIK3-knockdown plasmid. GAPDH was used as a loading control. **(C)** Western blot analysis of nuclear-YAP1, nuclear-TAZ and nuclear-TEAD1 in EFHD1-overexpressed-transfected HCT116 cells in the presence or absence of SIK3-knockdown plasmid. H3 was used as a loading control. **(D)** Immunofluorescence staining of YAP1 (red) for EFHD1-overexpressed-transfected HCT116 cells in the presence or absence of SIK3-knockdown plasmid. Nuclei were stained with DAPI (blue). Representative images were shown. Data were represented as mean ± SD. *p < 0.05, **p < 0.01 and ***p < 0.001 as compared to the control group.

To further investigate the regulatory mechanism of EFHD1 in the SIK3 signaling pathway, EFHD1-related molecules, as reported in the literature 9, were summarized and tested. Recent evidence suggests that the Hippo signaling pathway contributes to cancer development. Moreover, EFHD1 participates in the regulation of YAP1 phosphorylation, which is a key mediator of the Hippo signaling pathway. Therefore, we examined the effects of EFHD1 on the Hippo signaling pathway. Our results suggest that the protein expression levels of p-LATS1, p-YAP1, and p-TAZ were dramatically elevated by EFHD1 overexpression in HCT116 and SW480 CRC cells (**Figure 6A**). However, SIK3 knockdown significantly inhibited the expression of p-LATS1, p-YAP1, and p-TAZ and reduced the upregulation of p-LATS1, p-YAP1, and p-TAZ enhanced by EFHD1 (**Figure 6B**). Additionally, EFHD1 overexpression reduced nuclear YAP1, nuclear TAZ, and TEAD1 levels, thereby preventing YAP1/TAZ nuclear translocation and interaction with TEAD1, a key process in tumor progression. In contrast, SIK3 knockdown promoted YAP/YAZ translocation and TEAD1 expression (**Figure 6C**), as confirmed through immunofluorescence staining (**Figure 6D**). These results suggested that EFHD1 upregulates SIK3 to exert anti-cancer effects by activating the Hippo signaling pathway in CRC cells.

## Discussion

Metastasis is the primary cause of CRC mortality, with over 50% of patients diagnosed at varying stages of metastatic disease 18. Therefore, there is growing interest in identifying novel strategies to mitigate metastatic CRC. EFHD1, an EF-hand superfamily protein, has recently received considerable attention in cancer research 7-9, although its anti-cancer effect in CRC remains poorly understood. In this study, we found that EFHD1 exerted substantial inhibitory effects on colorectal carcinogenesis and metastasis by upregulating SIK3 through modulation of the Hippo signaling pathway. Our findings establish EFHD1 as a novel and promising therapeutic target for CRC prevention.

EFHD1 encodes a member of the EF-hand superfamily of calcium-binding proteins that are involved in a variety of cellular processes. Recently, the role of EFHD1 in tumor progression was revealed. The role of EFHD1 in tumor progression has been explored in gastric, breast, and clear-cell renal cancers 5, 8, 9. Notably, aberrant EFHD1 methylation has been observed in CRC, highlighting its potential clinical significance for CRC detection and treatment 19. Although the differential gene expression profile of EFHD1 between normal and CRC tissues can be obtained from the GEPIA database, the protein levels of EFHD1 in CRC have not been validated. In this study, EFHD1 expression in paracancerous tissue samples was noticeably higher than that in tumor tissue samples. In addition, the protein level of EFHD1 was lower in CRC cells than in normal colon cells. These findings verify that EFHD1 is dysregulated in CRC and that EFHD1 may be a new prognostic and therapeutic biomarker for the clinical management of CRC.

EMT is a complex biological process that mediates the interconversion between epithelial and mesenchymal cells. EMT occurs in various cancer types 20. It enables tumor cells to lose their adhesive properties and gain migratory and invasive capabilities, making it easier to detach from primary sites, enter the blood circulatory system, and spread to distant organs. EMT activation results in tumor cell survival, metastasis, immunosuppression, and poor prognosis 21. EMT is usually associated with the aberrant expression of calcium adhesion proteins (cadherins) 22. E-cadherin, a single-channel type I transmembrane classical cadherin, is widely expressed in various types of epithelial cells. It promotes the formation of epithelial tight junctions and inhibits abnormal cell proliferation to maintain epithelial structural stability. In contrast to E-cadherin, N-cadherin, another calcium-dependent transmembrane protein in the classical cadherin family, increases connection flexibility and facilitates cell mobility and separation. Loss of E-cadherin and elevation of N-cadherin are fundamental characteristics of malignant EMT and have become molecular markers for cancer diagnosis. E-cadherin expression significantly decreases, while N-cadherin expression increases, in liver, lung, breast, gastric, and colon cancers, leading to inhibited apoptosis, poor differentiation, and accelerated metastasis 23-27. Vimentin, an important intermediate filament protein in eukaryotes, plays a central role in the modulation of EMT signaling. It is primarily responsible for cytoskeleton construction and maintenance of cellular morphology, preventing protein misfolding in tumor cells. Vimentin is strongly and positively expressed in endometrial, ovarian, and colorectal cancers 28-30, providing diagnostic and therapeutic insights. Matrix metalloproteinases (MMPs), also known as gelatinases, degrade various extracellular matrix proteins 31. MMP2 and MMP9 are important MMPs involved in cell dispersion, differentiation, and angiogenesis. Inhibition of MMP2 and MMP9 has been shown to alleviate cancer progression 32.

In the present study, EFHD1 significantly suppressed CRC cell proliferation by downregulating MCM2 and PCNA expression. It also attenuated EMT by increasing E-cadherin levels while reducing the expression of N-cadherin, vimentin, MMP2, and MMP9 *in vitro*. Additionally, EFHD1 limited CRC tumor growth and lung metastasis *in vivo*. According to the GEPIA data platform, there is a strong positive correlation between EFHD1 and SIK3 expression in CRC tumors and normal colon tissues. The expression of SIK3 is much higher in normal tissues than in the tumor tissues of patients with CRC. SIK3 has been identified in various types of malignancies; however, little work has been done to investigate its regulatory abilities and molecular mechanisms in CRC. Our results demonstrate that EFHD1 upregulates SIK3, which mimics EFHD1's ability to inhibit EMT-related markers, including N-cadherin, vimentin, MMP2, and MMP9. Conversely, SIK3 knockdown increases the expression of these markers and partially reverses EFHD1's inhibitory effects on EMT. Thus, EFHD1 effectively suppresses CRC metastasis by upregulating SIK3 to inhibit EMT. Targeting the EFHD1-SIK3 axis may be an effective strategy for overcoming CRC cell proliferation and metastasis.

The Hippo signaling pathway, also known as the Salvador-Warts-Hippo pathway, has emerged as a critical focus in cancer research owing to its role in regulating cell division, apoptosis, and stem cell self-renewal 33. As a tumor-suppressive pathway, it restricts cell proliferation and promotes apoptosis, making it particularly relevant to cancers characterized by uncontrolled cell growth 34. According to previous reports, EFHD1 and SIK3 activate the Hippo signaling pathway by phosphorylating yes-associated protein-1 (YAP1), thereby preventing its nuclear translocation 9. However, the ability of EFHD1 and SIK3 to inhibit CRC via Hippo pathway activation remains unexplored. This study addresses this gap by investigating the regulatory effects of EFHD1 on SIK3 expression and the Hippo signaling pathway in CRC. The Hippo signaling pathway includes core kinase components such as large tumor suppressor kinase 1 (LATS1), YAP1, and a transcriptional coactivator with a PDZ-binding motif (TAZ) 35. LATS1, a key upstream enzyme in the Hippo signaling pathway, localizes to the mitotic apparatus and modulates the cell cycle. The N-terminal region and C-terminal kinase domain of LATS1 typically bind to cell cycle-related proteins to negatively regulate gene transcription. Notably, LATS1 is repressed in human cancers, and LATS1 mutations lead to soft tissue sarcoma formation 36. Downregulation of LATS1 phosphorylation triggers YAP1/TAZ nuclear transport, which induces EMT 37. YAP1, a highly active transcriptional coactivator of the Hippo signaling pathway, promotes the transcription of genes involved in the induction of cellular proliferation. TAZ, a close paralog of YAP1, is another major downstream effector of the Hippo signaling pathway associated with apoptosis suppression. Upon activation by LATS1, YAP1/TAZ phosphorylates its serine residues and remains inactive in the cytoplasm. However, dephosphorylation and hyperactivation of YAP1/TAZ are common in many malignancies 38. YAP1/TAZ, without cytoplasmic sequestration, readily localizes to the nucleus for transcription. As YAP1/TAZ lacks DNA-binding domains, it must bind to the TEA-domain family (TEAD) to perform its oncogenic activities. TEAD1, a nuclear transcription factor, contains bioactive DNA regions that interact with YAP1/TAZ. The TEAD-YAP1/TAZ complex is recognized as a tumor promoter that promotes cell regeneration, dispersion, and colonization 39. Thus, directly dissociating the TEAD-YAP1/TAZ complex may be an efficient means for interfering with TEAD-YAP1/TAZ-induced oncogenic events. In the present study, we found that EFHD1 overexpression promoted the phosphorylation of p-LATS1, p-YAP1, and p-TAZ, effectively preventing YAP1/TAZ from entering the nucleus and interacting with TEAD1. However, SIK3 knockdown reduced the phosphorylation of p-LATS1, p-YAP1, and p-TAZ, partially reversing EFHD1's effects on the Hippo signaling pathway. Taken together, the newly identified EFHD1-SIK3 axis participates in the modulation of the Hippo signaling pathway and serves as a novel regulator of CRC progression.

**Figure 7:**
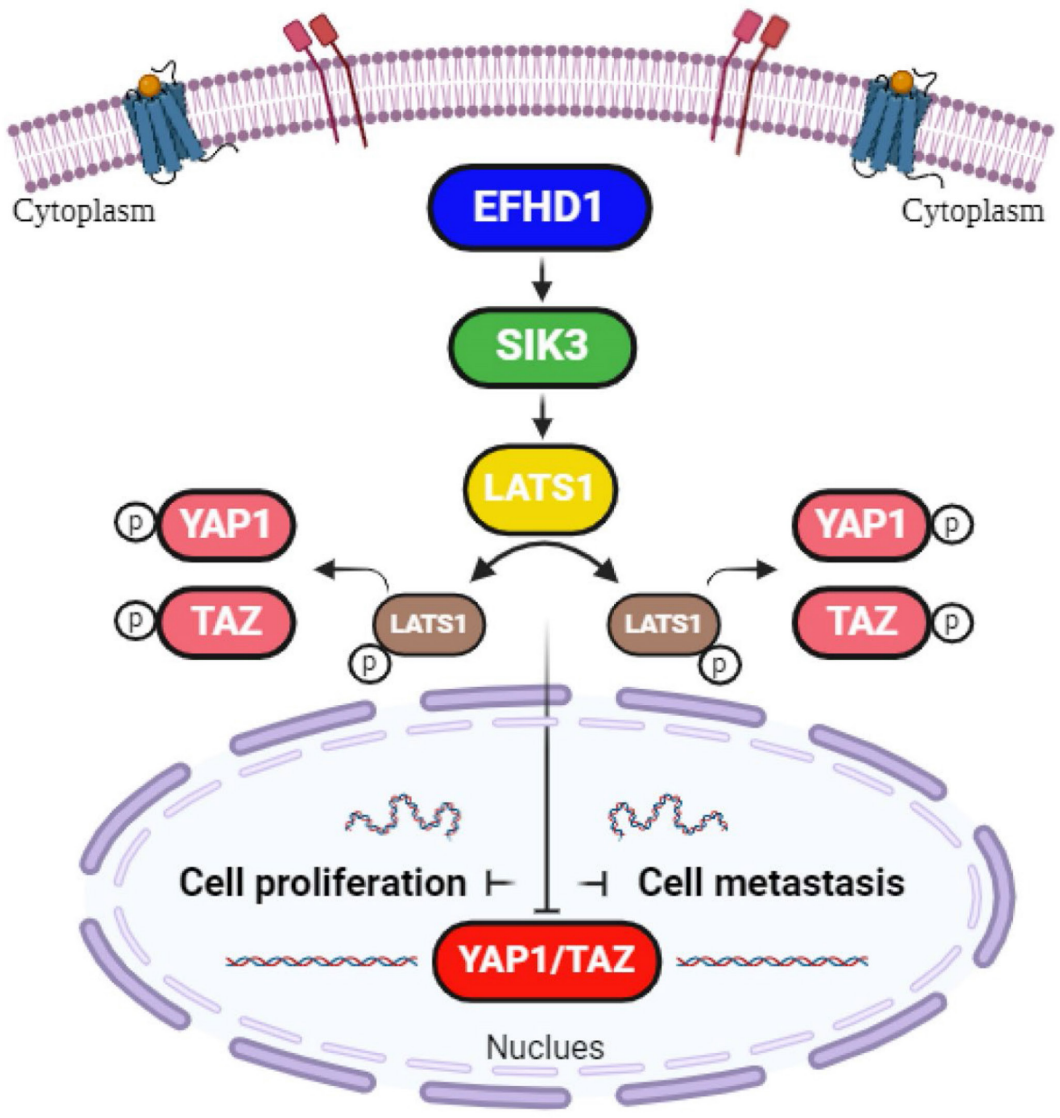
** Hypothetical model.** EFHD1 possesses potent anti-proliferative and anti-metastatic effects on CRC, the underlying mechanisms involve the upregulation of SIK3 through modulating Hippo pathway.

In conclusion, our study provides promising evidence that EFHD1 exerts potent antiproliferative and anti-metastatic effects in CRC, both *in vitro* and *in vivo*, by upregulating SIK3 to suppress EMT through modulation of the Hippo signaling pathway (**Figure 7**). Despite its promising potential, EFHD1 has yet to be explored for clinical anti-tumor applications. As a novel and compelling target, EFHD1 warrants further investigation as a therapeutic option for CRC. Future research should focus on identifying EFHD1 agonists, which could serve as an innovative strategy for CRC treatment.

## Abbreviations

Cadherins: calcium adhesion proteins
CRC: Colorectal cancer
EFHD1: EF-hand domain-containing protein D1
EMT: Epithelial-mesenchymal transition
IC_50_: 50% Inhibitory concentration
IHC: Immunohistochemical
IF: Immunofluorescence
LATS1: Large tumor suppressor kinase 1
MMP: Metalloproteinase
SIK3: Salt inducible kinase 3
TAZ: Transcriptional co-activator with PDZ-binding motif
TEAD: TEA-domain
YAP1: Yes-associated protein-1
